# Early Postural Changes in Individuals with Idiopathic Parkinson's Disease

**DOI:** 10.1155/2015/369454

**Published:** 2015-04-01

**Authors:** Mohamed Elsayed Khallaf, Eman Elsayed Fayed

**Affiliations:** ^1^Department of Physical Therapy for Neuromuscular Disorders and Its Surgery, Faculty of Physical Therapy, Cairo University, 7 Ahmed El-Zayat Street, P.O. Box 12611, Dokki, Giza, Egypt; ^2^Department of Physical Therapy, College of Applied Medical Sciences, University of Hail, P.O. Box 2440, Baqaa, Hail, Saudi Arabia

## Abstract

*Background and Objectives*. Postural changes are frequent and disabling complications of Parkinson's disease (PD). Many contributing factors have been evident either related to disease pathology or to adaptive changes. This study aimed at studying the postural changes in subjects with Parkinson's disease and its relation to duration of illness and disease severity.* Methods*. Eighteen patients with PD and 18 healthy matched volunteers represented the sample of the study. The patients were at stage 1 or 1.5 according to the Modified Hoehn and Yahr Staging with duration of illness between 18 and 36 months. Three-dimensional analysis of the back surface was conducted to explore the postural changes in the sagittal and frontal planes in both the patients and the healthy subjects.* Results*. Kyphotic angle, lordotic angle, fleche cervicale, fleche lombaire, scoliotic angle, and associated vertebral rotation and pelvic obliquity were significantly increased in patients with PD compared to the healthy subjects (*P* ≤ 0.05). There was no association between the measured postural changes and duration of illness as well as the severity of the IPD (*P* ≤ 0.05).* Conclusion*. Postural changes start in the early stages of idiopathic PD and they have no relationship to the duration of illness and disease severity.

## 1. Introduction

Patients with idiopathic Parkinson's disease (IPD) often present with abnormal posture [1]. In this population, posture may be affected in its orientation component (stooped posture, camptocormia, and Pisa syndrome) or in its balance component (loss of postural reflexes) [2]. The postural abnormality associated with kyphosis can cause chronic dysfunctions of the vertebral column or the surrounding structures. In later stages of IPD, it also disturbs standing and gait by affecting balance [3]. Impaired balance and falls are major sources of disability and decreased quality of life [4]. Moreover, 20% of patients with IPD may experience deep vein thrombosis and sudden death as a result of postural changes [5].

PD motor disorders are very complex and interconnected [6]. Patients might not complain about their abnormal posture until it interferes with their mobility or vision, especially if onset of the deformity was gradual [2, 7, 8]. Neurological examination often reveals marked axial rigidity [6, 9]. The paraspinal muscles may have a wooden consistency, and the rectus abdominis often feels tense [10]. Additionally, patients with IPD have an impaired body orientation with respect to gravity and are unable to maintain the vertical head and trunk orientation without vision [11].

There are many reports postulating that patients with early IPD can overcome deformity when asked to stand up straight in addition to a well-planned exercise therapy based on external cues and motor relearning principles [12, 13].

The control of postural stabilization has been widely studied at both the overall [14] and segmental levels [15], probably because falling has such serious effects on the daily life of Parkinsonian patients. Little attention has been paid so far, however, to earlier stages of IPD at which the postural changes might start. Additionally, very few studies investigated postural changes that might occur in patients with hemi-Parkinson's disease and its relation to duration of illness or disease severity.

In the present study, the specific objective is to measure the postural changes in subjects with hemi-Parkinson's disease. We measured the posture changes in the sagittal plane including kyphosis and lordosis as well as fleche cervicale and fleche lombaire which give the distance of the apex of the cervical and lumbar lordosis from a virtual vertical plumb line, yielding a fairly good approximation of the extent of thoracal kyphosis and lumbar lordosis [16]. The postural changes in the frontal plane, including pelvic obliquity, and scoliotic angle and associated vertebral rotation in individuals with IPD and healthy matched group were also studied. We hypothesized that there are significant differences between the idiopathic hemi-Parkinsonian patients and healthy subjects in postural orientation. The relationship between duration of illness, disease severity, and IPD postural changes was also studied. We hypothesized that there is no relation between duration of illness, severity according to Unified Parkinson's disease rating scale (UPDRS), and IPD postural changes and they are related to disease pathology.

## 2. Subjects and Methods

Eighteen patients with IPD were recruited from King Khalid Hospital, Hail, Saudi Arabia. In addition, 18 healthy age and sex matched volunteers participated in this trial. The duration of illness of the patients was between 18 and 36 months. The justification of the duration of illness is to avoid the effects of chronic adaptive musculoskeletal changes. Criteria for inclusion are documented positive response to chronic administration of Levodopa and typical clinical signs corresponding to UK brain bank criteria of IPD. The patients must be at stage 1 or 1.5 according to the Modified Hoehn and Yahr Staging. According to the UPDRS, subjects should earn a motor score of 14–19, activities of daily living score of 13–19, a walking score of 1-2, and a bradykinesia score of 1-2 during the on period. Subjects with history or clinical signs of peripheral neuropathy, spasticity, limb ataxia, symptomatic orthostasis, or dyskinesias affecting rasterstereographic recordings were not included. Additionally, patients who have secondary Parkinsonism or a rigidity score greater than 3 according to UPDRS were also excluded from the study. The study was approved by the Institutional Ethics Committee and a written informed consent was obtained from each participant.

**Figure 1:**
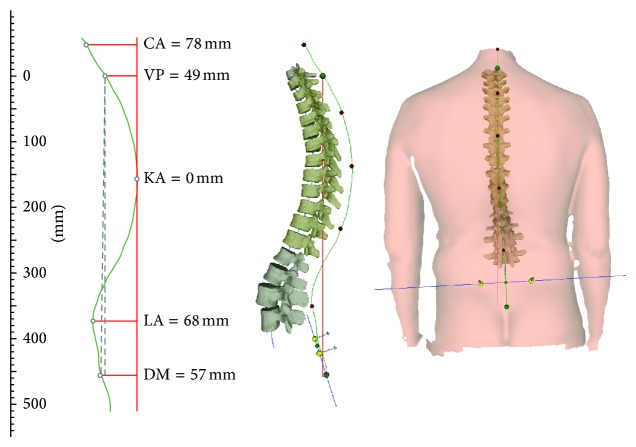
3-dimensional analysis of the back: postural changes in the sagittal (kyphosis) and frontal planes (scoliosis, coronal imbalance, and pelvic obliquity). CA: cervical Apex, VP: vertebral promines, KA: kyphotic apex, LA: lumber apex, DM: Dimple Middle.

Three-dimensional analysis of the back surface was conducted with the rasterstereographic device formetric 4D (Diers International GmbH, Schlangenbad, Germany). Rasterstereography allows a contactless and radiation-free determination of the body surface [17]. In rasterstereographic measurements, parallel white light lines are projected on the back surface of the examinee. The three-dimensional back shape leads to a deformation of these lines, which can be detected by a camera. Anatomical landmarks are hereby automatically captured by assigning concave and convex areas to the curved light pattern [18]. With these anatomical fix points, the system is able to calculate a three-dimensional model of the human spine and clinically relevant parameters, such as the kyphosis angle (measured between C7 and the thoracic-lumbar inflection point) and lordosis angle (measured between the surface tangents of the thoracic-lumbar inflection point ITL and the lower lumbar-sacral inflection point) of the spine, can be determined. The two lumbar dimples (dimple left (DL) and dimple right (DR)) are in close relation to the underlying posterior superior iliac spines [19]. Therefore, it is possible to use them to determine pelvic obliquity. Fleche cervicale is horizontal spatial distance between the plumb line and cervical apex (mm). Fleche lombaire is the horizontal spatial distance between the plumb line and lumbar apex (mm) (Figure 1).

For the measurements, the participants were undressed down to the buttocks and the neck is exposed up to the hairline. Rings and watches were removed in order to prevent the light grid from being reflected on the one hand and so that no artificial changes are created on the other hand. The patients were examined in a relaxed, standing position with the back facing the system, and arms are hanging below the body; shoulders are in neutral position. To standardize the foot positioning the participants were directed to stand with their feet against specifically placed metal bar. Once the patient is displayed on the monitor, the camera height can be adjusted. The height is set when the patient is relatively centered on the monitor and the green horizontal line is at the level of the inferior angle of the scapula. After back scanning, the accuracy of the anatomical landmarks VP (vertebra prominens, C7), SP (sacrum point), and the two lumbar dimples DL/DR (dimple left/dimple right) has been verified and are in the right point. Measurement was repeated if the aforementioned landmarks have been located incorrectly on all images.

### 2.1. Statistical Analysis

The data has been analyzed using the SPSS software version 17 (SPSS Inc., Chicago, IL, USA). Descriptive statistics were calculated to summarize the demographic data of the healthy subjects and IPD patients. This demographic data was compared between groups using *t*-test (*P* ≤ 0.05). Mean and standard deviation were used to figure out the clinical characteristics of the IPD patients. The pelvic obliquity, kyphotic angle, lordotic angle, fleche cervicale, fleche lombaire, scoliotic angle, and maximum vertebral rotation were compared between groups using *t*-test with level of significance set at *P* ≤ 0.05. Pearson correlation analyses were performed to assess the association between postural changes and duration of illness as well as the severity of the disease.

## 3. Results

The demographic characteristics of the participants are detailed in Table 1. There were 18 patients with a diagnosis of unilateral IPD and 18 healthy subjects. The healthy subjects and Parkinsonian patients were matched for age (*P* = 0.20), body weight (*P* = 0.39), body height (*P* = 0.59), and body mass index (*P* = 0.51). Clinical examination of the participants revealed that the IPD patients have a mean duration of illness of 35.57 ± 4.08 months. The motor score of the UPDRS was 16.43 ± 2.23 while the activity of daily living score was 13.57 ± 1.72 (Table 2). According to Hoehn and Yahr classification, the participants were staged as belonging to Class 1 in 7 patients and Class 1.5 in 11 patients with a mean of 1.29 ± 0.26.

**Figure 2:**
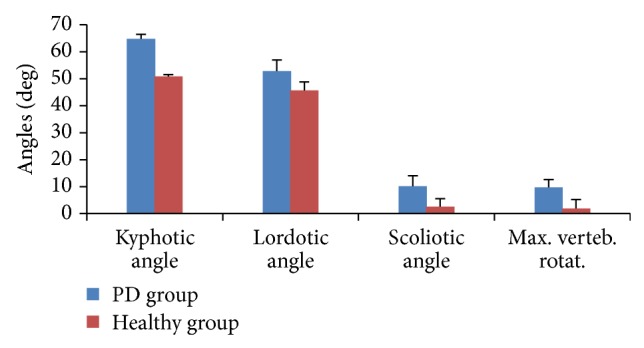
Rasterstereographic analysis of patients and healthy subjects. Significant differences were found in the kyphotic, lordotic, and scoliotic angles and maximum vertebral rotation.

**Figure 3:**
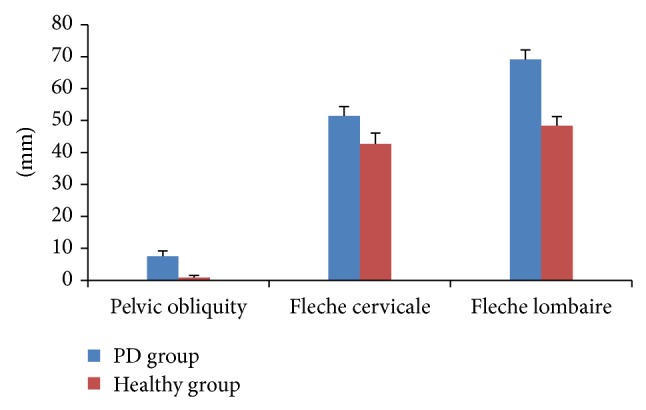
Rasterstereographic analysis of patients and healthy subjects. Significant differences were found in the pelvic obliquity, fleche cervicale, and fleche lombaire.

Paired *t*-test was used for enlightening the differences between groups (Figures 2 and 3). In the sagittal plane, kyphotic angle, lordotic angle, fleche cervicale, and fleche lombaire were significantly different among the healthy subjects and patients with IPD (*P* ≤ 0.05). These significant differences indicate early changes in IPD patients' posture. The mean kyphotic angle among the IPD patients was 64.86 ± 4.14° and 50.86 ± 3.13° among healthy subjects. A significant difference in the kyphotic angle was found with *t* = 11.913 and *P* = 0.001. The measured lordotic angle among IPD patients was 52.86 ± 3.89° and 45.71 ± 2.98° was recorded from the healthy subjects. Statistical analysis showed the existence of a significant difference between the two measures of the IPD and the healthy subjects (*t* = 4.656 and *P* = 0.003). Fleche cervicale and fleche lombaire were 42.71 ± 3.35 mm and 48.43 ± 2.82 mm, respectively, in the healthy subjects; on the other hand they recorded 51.43 ± 2.94 mm and 69.14 ± 2.97 mm, respectively, among IPD participants. The significant differences in fleche cervicale and fleche lombaire ensure the early postural changes in the sagittal plane (Figures 2 and 3).

Scoliotic angle and associated vertebral rotation as well as pelvic obliquity have been significantly different in individuals with hemi-Parkinsonian patients compared to healthy matched group. The measured pelvic obliquity among the healthy subjects was 0.86 ± 0.69 mm and 7.57 ± 1.62 mm was recorded from the IPD group (*t* = 12.871 and *P* = 0.001). Comparisons between the scoliotic angle measured in IPD patients (10.14 ± 0.38°) and healthy group (2.57 ± 1.13°) showed significant differences (*t* = 14.55 and *P* = 0.002). In the same context, a significant difference between patients (9.57 ± 1.13) and healthy (1.86 ± 0.69) subjects was found in regard to the vertebral rotation (*t* = 6.827 and *P* = 0.001) (Figures 2 and 3).

The duration of illness (years from diagnosis) was nonsignificantly correlated with the overall sagittal and frontal plane postural changes (Table 3) including pelvic obliquity (*P* = 0.596), lordotic angle (0.824), fleche cervicale (*P* = 0.279), fleche lombaire (*P* = 0.733), scoliotic angle (*P* = 0.480), and maximum vertebral rotation (*P* = 0.835). Moreover, the motor score of UPDRS was not significantly correlated with the overall sagittal and frontal plane postural changes (Table 3) including pelvic obliquity (*P* = 0.459), lordotic angle (0.755), fleche cervicale (*P* = 0.353), fleche lombaire (*P* = 0.222), scoliotic angle (*P* = 0.113), and maximum vertebral rotation (*P* = 0.204).

## 4. Discussion

Given the high risk of short-term and long-term complications affecting individuals with Parkinson's disease, the current research presents the analysis of posture in early stages that may help to early identify, prevent, or delay progression of postural disorders. Additionally, this will improve rehabilitation strategies and conservative management of typical trunk neurogenic deformities detected in patients affected by IPD. Our results support the previously stated hypothesis that postural changes start in the early stages of IPD. In the sagittal plane, kyphotic angle, lordotic angle, fleche cervicale, and fleche lombaire were significantly different among the healthy subjects and patients with IPD. Increased kyphotic and lordotic angles, fleche cervicale, and fleche lombaire could be the consequence of axial rigidity of the flexor muscles with weakness of the erector spinal muscles due to disturbed motor programming and the sensory control of motor programs within the basal ganglia. An imbalance between excessive central motor drive to the ventral and dorsal trunk musculatures (leading to excessive activation of the abdominal wall muscles) and reduced motor drive to the paraspinal muscles (which would favour secondary muscle atrophy and injury) may also contribute to this abnormal posture [20]. In patients with PD, there is continual abnormal muscle recruitment and activation with constant use of the axial muscles to maintain posture [21]. Additionally, patients with PD respond abnormally to perturbations during stance showing reduced intersegmental flexibility [14, 22] which may cause reduced range of spinal movements especially around the spinal axis [23, 24]. These deficits might be compensated by flexion in the sagittal or coronal planes to maintain the centre of gravity within the limits of stability and to prevent falls. Our results are also in close agreement with Lepoutre and colleagues who found that lack of use of the erector muscles due to rigidity of the spinal flexors could induce a fatty involution in the paraspinal muscle region. Subsequently, vertebral deformations could appear [11].

Similarly scoliotic angle and associated vertebral rotation as well as pelvic obliquity have been significantly increased in individuals with hemi-Parkinsonism compared to healthy matched group. We also found that scoliosis contralateral to the side of initial symptoms is significantly obvious compared to ipsilateral scoliosis. This can be attributed to impaired ability determining body position in space, impaired proprioception that was found in patients with hemi-Parkinsonism and rigidity of lateral trunk flexors. This is consistent with Tagliabue et al., who emphasized the importance of an intact basal ganglion and proprioceptive feedback for the precision and coordination of the posture [25]. Additionally, it was reported that there is hyperactivity of paravertebral muscles contralateral to the leaning side [26]. Based on our results, it was clear that postures of all Parkinsonian patients shared in the study are complex, not only for lateral flexion, but also for forward flexion. All of the patients were not themselves aware of their abnormal postures. The forward flexion is established by increased kyphotic angle, fleche cervicale, and fleche lombaire. Additionally the lateral flexion is inveterated by a significantly increased scoliotic angle and vertebral rotation in addition to the pelvic obliquity.

Furthermore, in contrast with previously reported data [26, 27], the lack of correlation with duration of illness and disease severity indicates that the modification of these postural parameters likely represents a typical pathological adaptation of the spine to the disease [28] and may raise a question about the relationship between the postural changes and duration of illness in different clinical phenotypes of PD.

An important limitation of this study was the small sample size. This can be attributed to the limited number of population in Hail, Saudi Arabia, and decreased number of IPD referred to physical therapy in the early stages. Nevertheless, we were able to show that the postural changes start early in cases of IPD which open the door to the prevention measures and rehabilitation researches to delay or postpone the resulting complications.

## 5. Conclusion

Postural changes start in the early stages of idiopathic Parkinson's disease and they have no relationship to the duration of illness and disease severity.

## Figures and Tables

**Table 1:** Demographic data of the participants.

	PD group	Healthy group	*P*
Age (yrs)	56.14 ± 2.34	55.71 ± 2.21	0.20
Sex (M/F)	10/8	11/7	0.33
Height (m)	1.78 ± 0.05	1.76 ± 0.07	0.59
Weight (kg)	81.86 ± 5.96	79.71 ± 7.18	0.39
BMI	25.93 ± 0.88	25.70 ± 0.95	0.51

*P* is significant at *P* ≤ 0.05. M: males; F: females.

**Table 2:** Clinical characteristics of PD patients.

Characteristic	Mean ± SD
Duration of illness (m)	35.57 ± 4.08
MHY stage	1.29 ± 0.26
Motor score_(UPDRS)_	16.43 ± 2.23
ADL score_(UPDRS)_	13.57 ± 1.72
Walking_(UPDRS)_	1.29 ± 0.49
Bradykinesia_(UPDRS)_	1.71 ± 0.49
Rigidity_(UPDRS)_	1.57 ± 0.53
Levodopa alone (mg)_(6/18)_	275 ± 37.9
Levodopa and pramipexole (mg)_(12/18)_	300 ± 41.6–1.08 ± 0.67
Side at onset (Rt)	83.3%
Side of lateral flexion (Rt)	22.2%

UPDRS: Unified Parkinson's Disease Rating Scale; ADL: activities of daily living; MHY: Modified Hoehn and Yahr; Rt: right.

**Table 3:** Correlation coefficients between duration of illness, UPDRS motor score, and postural parameters.

		Pelvic obliquity	Kyphotic angle	Lordotic angle	Fleche cervicale	Fleche lombaire	Scoliotic angle	Max. vertebral rotation
Duration of illness	Pearson correlation	0.245	0.104	0.363	0.477	−0.159	−0.323	0.098
	*P*	0.596	0.824	0.423	0.279	0.733	0.480	0.835

UPDRS (motor)	Pearson correlation	0.338	−0.741	0.146	−0.417	−0.529	0.651	−0.820
	*P*	0.459	0.057	0.755	0.353	0.222	0.113	0.204
